# Genome Sequence of *Arthrobacter* sp. Strain ATA002, a Seed Endophytic Bacterium from the Atacama Desert

**DOI:** 10.1128/mra.00027-23

**Published:** 2023-04-10

**Authors:** Rómulo Oses Pedraza, Julia Mitzscherling, Daniel Lipus, Dirk Wagner, Alexander Bartholomäus

**Affiliations:** a Centro Regional de Investigación y Desarrollo Sustentable de Atacama (CRIDESAT), Universidad de Atacama, Copiapó, Chile; b GFZ German Research Centre for Geosciences, Geomicrobiology Section, Potsdam, Germany; c University of Potsdam, Institute of Geosciences, Potsdam, Germany; Indiana University, Bloomington

## Abstract

The Gram-positive diazotrophic seed endophytic bacterium *Arthrobacter* sp. strain ATA002 was isolated from seeds of the endemic cactus Maihueniopsis domeykoensis collected in the Atacama Desert, Chile. Here, we present a circular genome sequence, obtained by Nanopore sequencing, with a size of 3,904,590 bp and a GC content of 65.9%.

## ANNOUNCEMENT

The Atacama Desert is one of the most extreme environments on Earth (1). It is characterized by soils with low levels of nitrogen and nutrients, extreme temperature oscillations, high levels of UV radiation, and very low annual precipitation. Plants living in this environment need unique adaptations to these harsh conditions. Seed endophytic bacteria (SEB) can increase plant fitness by inducing or modulating the plant gene expression related to growth, development, and defense (2, 3). SEB are believed to influence the development of the plant. They can be transmitted to the next generation and become the first colonizers of the roots and shoots of the seedlings after germination (4).

Seeds of Maihueniopsis domeykoensis were collected by AELA SpA and supplied by the Forestry Institute - INFOR (La Serena, Chile) at Domeyko (−28.952722 N, −70.894150 E; located between the cities of La Serena and Vallenar). The surface of the seeds was sterilized by sequential washing in 50% ethanol for 1 min, 2% sodium hypochlorite for 3 min, and 50% ethanol for 30 s, rinsing the seeds twice with sterile distilled water, grinding them in a clean mortar, and suspending them in combined carbon or Rennie semi-solid N-free medium (5) for 24 h at 30°C. After 72 h of incubation on N-free agar (NFA) at 30°C, individual bacterial colonies were isolated and purified on new NFA. Genomic DNA was extracted from a pure culture grown on LB medium at 28°C using the UltraClean microbial DNA isolation kit (MoBio, Carlsbad, CA, USA). All incubation and culturing steps took place under aerobic conditions.

High-molecular-weight DNA was prepared without specific size selection using the rapid sequencing kit (Oxford Nanopore Technologies [ONT], Oxford, UK). The DNA was cleaned using AMPure XP beads (Beckman Coulter, Pasadena, CA). The library was sequenced using the MinION platform and the Flongle flow cell (ONT). The sequencing ran for 72 h, and the quality was monitored using the MinKNOW v22.05.5 interface (ONT). Default parameters were used for all software unless otherwise specified. The raw sequencing data were base called and demultiplexed with super high accuracy using Guppy v6.0.6+8a98bbcbd (ONT), resulting in 100,206 reads with an *N*_50_ value of 4,851 bp. Assembly, the first round of polishing, and circularity assessment were performed using Flye v2.9.1-b1780 (6) (parameters, –meta –nano-raw). A second round of polishing was performed using Medaka v1.7.2 (ONT). The genome quality was assessed and full-length 16S rRNA sequences were recovered using CheckM v1.2.1 (7). The genome characteristics were assessed using QUAST v5.2.0 (8).

The resulting genome was a circular contig 3,904,590 bp long, with a GC content of 65.9% and a coverage of 59×. The genome was found to be 94.15% complete and 0.98% contaminated. The taxonomic affiliation and closest related species were inferred using GTDB-Tk v2.1.0 (9), confirming that the isolate belongs to the genus *Arthrobacter*. The closest genome was Arthrobacter gandavensis strain JCM 13316 (average nucleotide identity [ANI], 84.82%; GenBank accession number GCF_009192735.1). A full-length 16S rRNA sequence comparison using NCBI BLAST (10) revealed the highest identity of 98.89% with Arthrobacter koreensis strain DL (CP106856.1), with a query coverage of 99%. The genome was annotated using the NCBI PGAP v6.3 pipeline (11).

### Data availability.

The genome of *Arthrobacter* sp. ATA002 was deposited at GenBank under the accession number CP114015 and the BioProject accession number PRJNA894817. The raw reads are accessible via the SRA accession number SRR22099512.
